# Supplementary material for: Small molecule inhibitors of 15-PGDH exploit a physiologic induced-fit closing system
Source: Nat Commun. 2023 Feb 11;14:784. doi: 10.1038/s41467-023-36463-7 (PMC9922282; doi:10.1038/s41467-023-36463-7)
Supplement: Supplementary file 1 — Supplementary Information [file 41467_2023_36463_MOESM1_ESM.pdf]

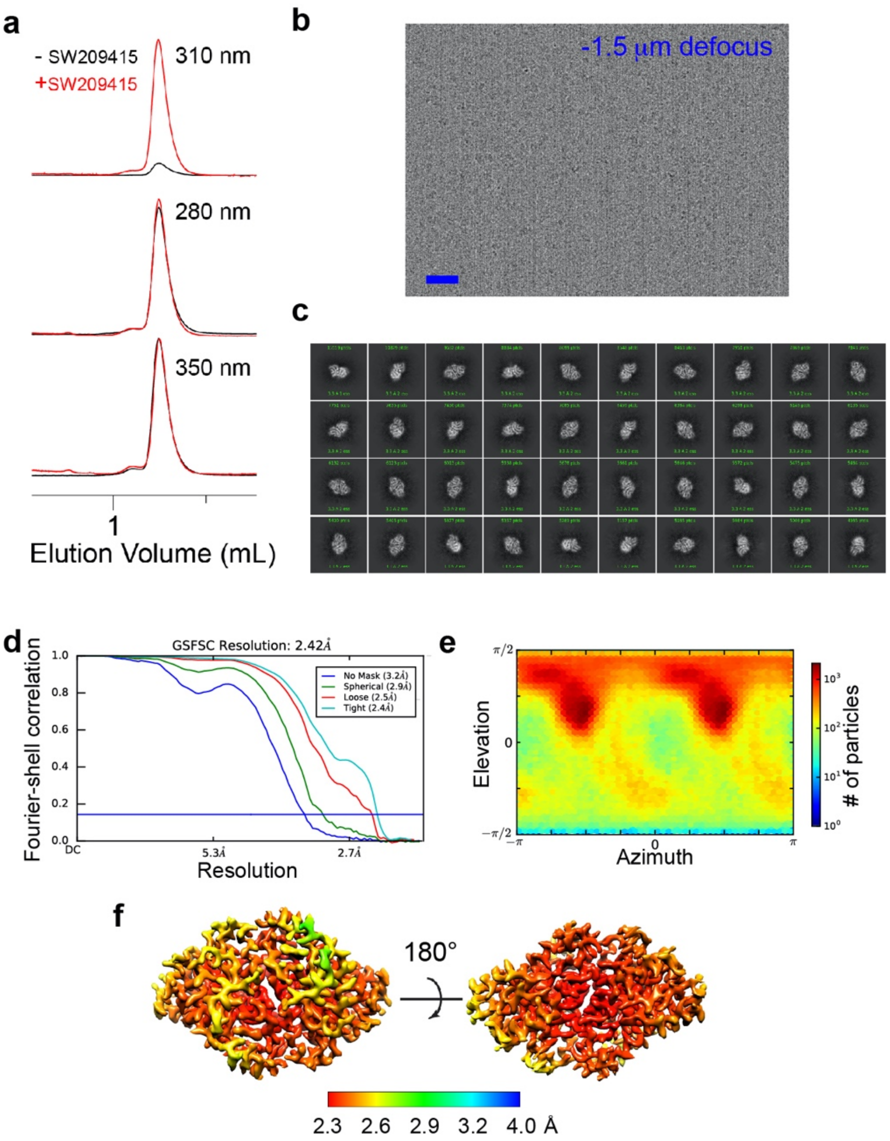

Supplementary Fig. 1 | Cryo-EM data processing of 15-PGDH-NADH-SW209415 complex. **a,** Size-exclusion chromatography profiles of 15-PGDH with (red line) and without (black line) (+)-SW209415. UV absorbance at 310 nm was used to monitor (+)-SW209415 and 280 nm was used to monitor 15-PGDH protein in the chromatogram. **b,** Representative cryo-EM image of human 15-PGDH bound with (+)-SW209415 collected on a Gatan K3 camera with CDS mode. Scale bar is 500 Å. **c,** Representative 2D class averages of 15-PGDH-inhibitor complex with different projection views. **d,** GSFSC curve of the final reconstruction of 15-PGDH-NADH-SW209415 complex showing an overall resolution of 2.4 Å using FSC at 0.143. **e,** Angular distribution of particles from the final 3D reconstruction. **f**, Local resolution of 15-PGDH-NADH-SW209415 complex shown in color-coded map.

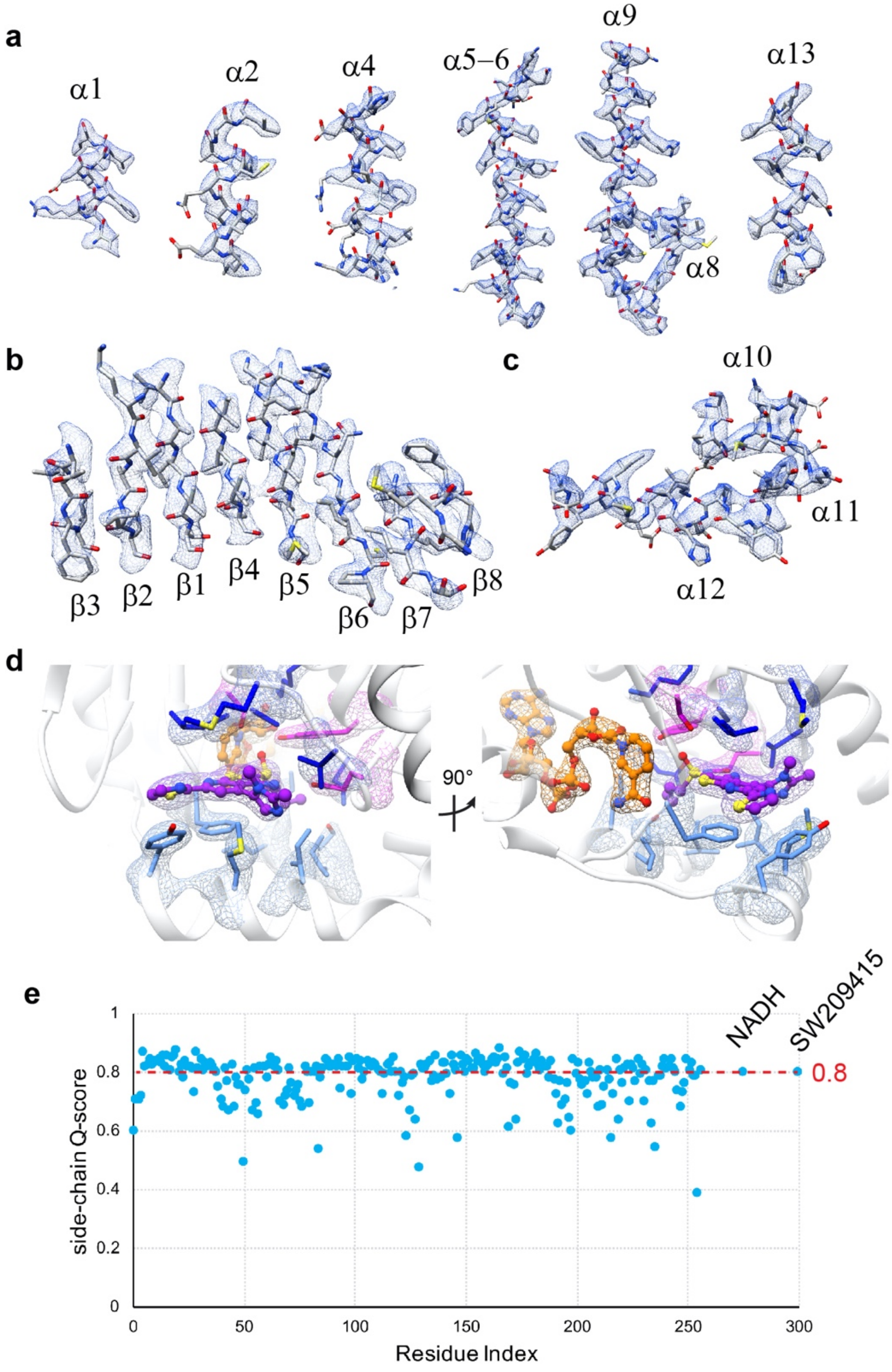

Supplementary Fig. 2 | Cryo-EM density map and refined structure human 15-PGDH-NADH-SW209415 complex. Cryo-EM density map and model are shown for **a-c,** α-helices and β-strands of human 15-PGDH. **d,** Bound (+)-SW209415 and NADH shown in purple and orange, respectively, with corresponding mesh density. **e,** *Q-score* value per residue of the 15-PGDH with (+)-SW209415 cryoEM model and map. The median value of Q-score is 0.8.

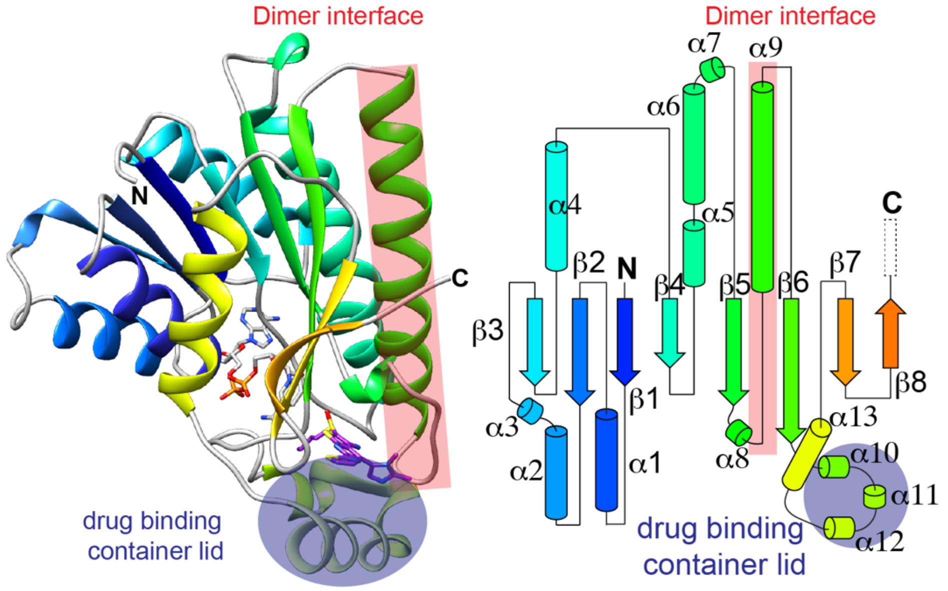

Supplementary Fig. 3 | Schematic diagram of human 15-PGDH monomer. The homodimeric interface and the lid of drug binding pocket are highlighted in red rectangle and blue circle, respectively.

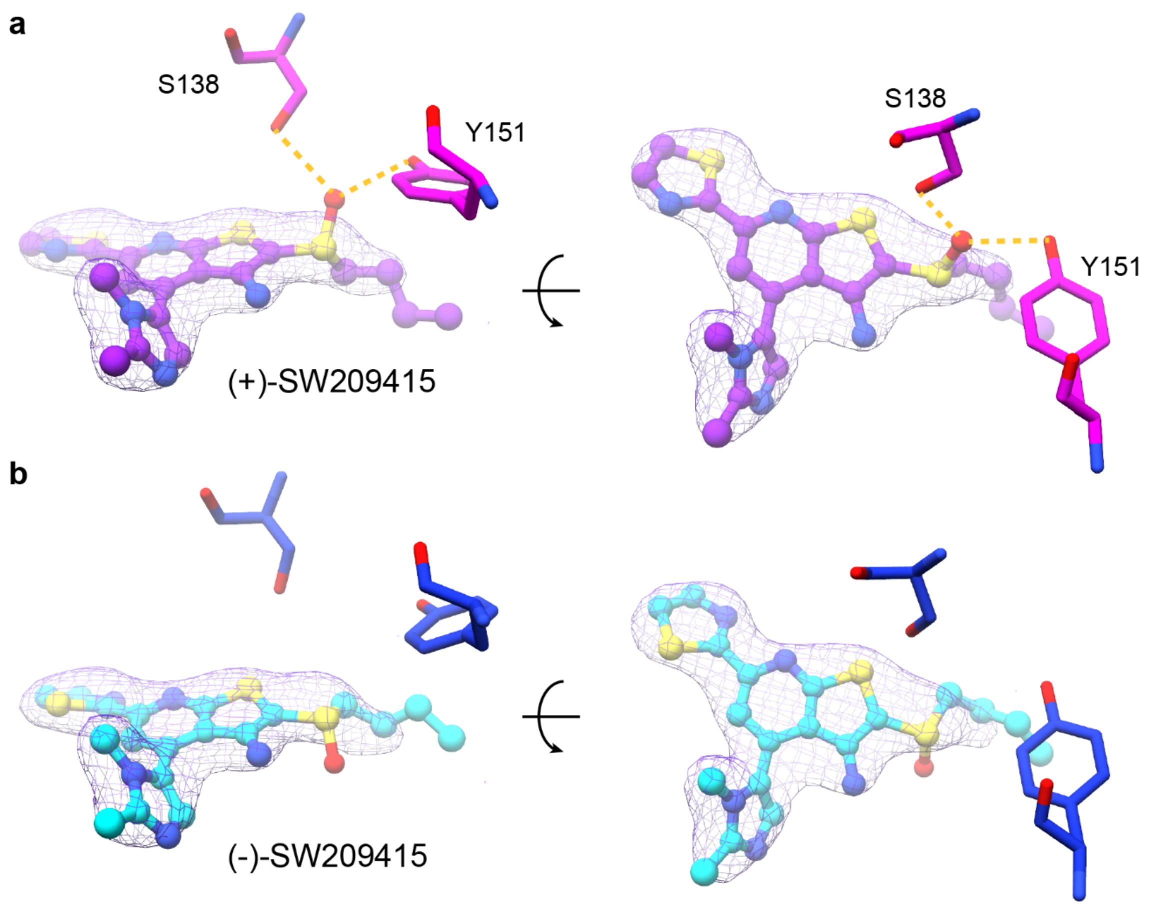

Supplementary Fig. 4 | Enantiomer specific binding of SW209415 to 15-PGDH. **a,** Docking of (+)-SW209415 and **b,** (-)-SW209415 enantiomers into the cryo-EM density of 15-PGDH-NADH-SW209415 complex. Docking of the enantiomers into the cryo-EM density shows that the (+) enantiomer uniquely fits into the electron density and forms favorable hydrogen bonds with the residues in the catalytic center of 15-PGDH.

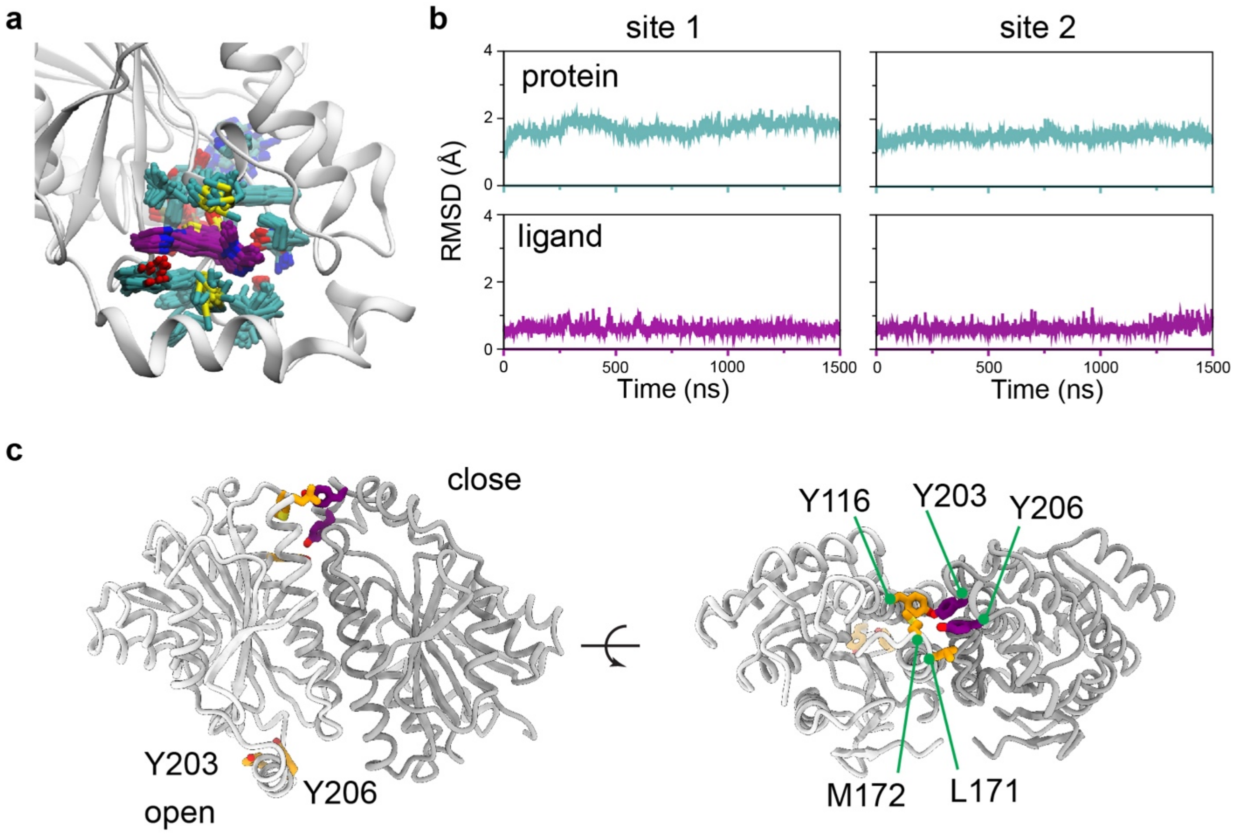

Supplementary Fig. 5 | Molecular dynamic (MD) simulations validate the binding pose of (+)-SW209415 with 15-PGDH. **a,** Superposition of snapshots (every 0.1 μs) from a 1.5 μs trajectory starting with the structure determined by cryo-EM. The simulation shows only minor changes in inhibitor or protein structure over this timescale. **b,** Time evolution of root-mean-square-deviation (RMSD) of protein and ligand shows a low and stable RMSD value for (+)-SW209415 and 15-PGDH protein for both protomers over the course of the simulations. **c,** A snapshot from the trajectories of the NADH-bound form of 15-PGDH illustrating the asymmetric dynamic of the two container lids. Residues (purple) from one lid make contacts with the dimeric interface (orange) of the opposite protomer as labeled and shown in stick representation.

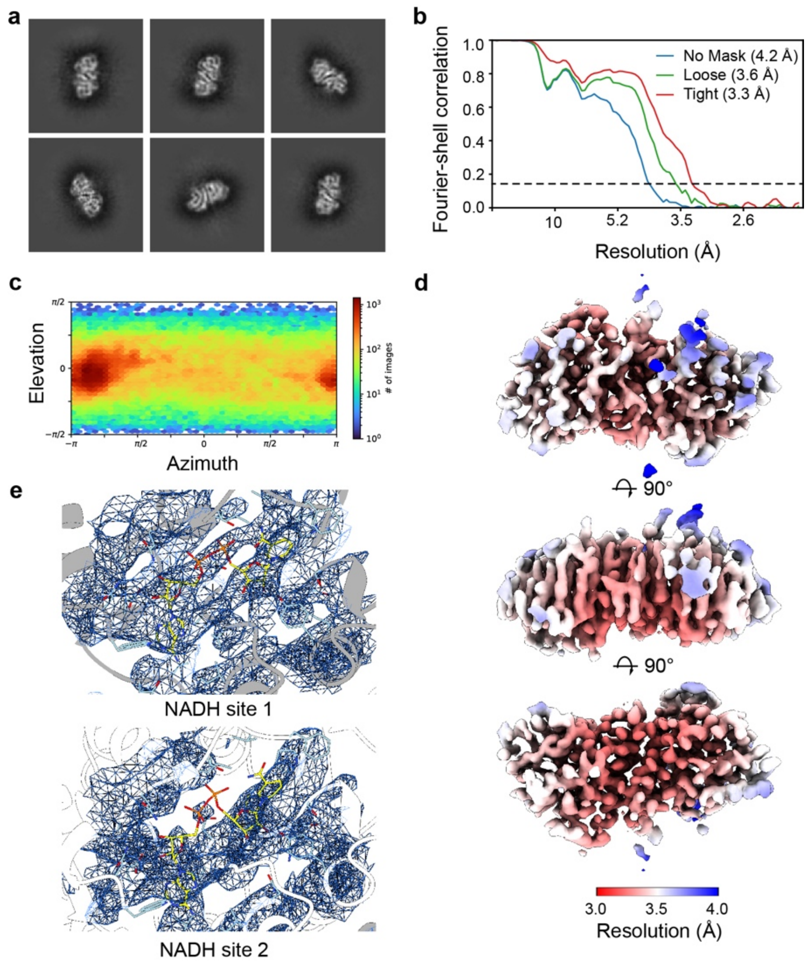

Supplementary Fig. 6 | Cryo-EM data processing of 15-PGDH with NADH. **a,** Representative 2D class averages of 15-PGDH-NADH with different projection views. **b,** GSFSC curve of the final reconstruction of 15-PGDH-NADH complex showing an overall resolution of 3.3 Å using FSC at 0.143. **c,** Angular distribution of particles from the final 3D reconstruction. **d**, Local resolution of 15-PGDH-NADH complex shown in color-coded map. **e**, Cryo-EM density of cofactor NADH observed in each 15-PGDH protomer. Site 1 has strong density accounting for the cofactor, while site 2 of the cryo-EM map has weaker features for the nicotinamide and diphosphate groups. Map displayed at a contour level of 0.1.

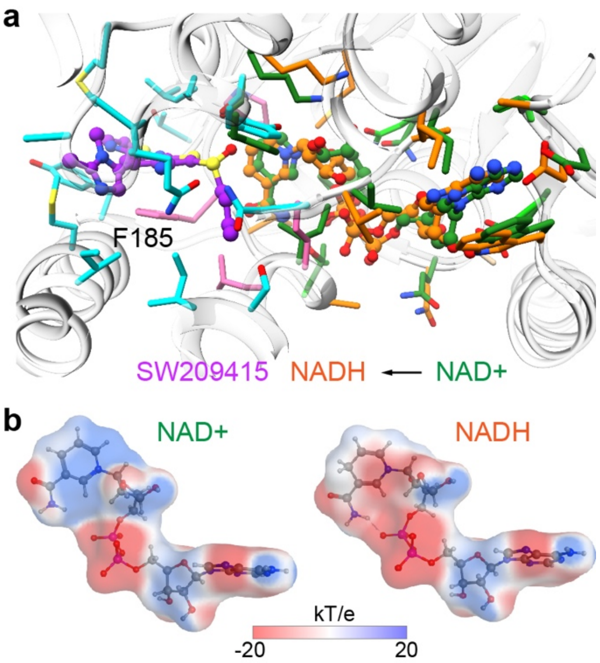

Supplementary Fig. 7 | Comparison of NAD+ and NADH bound 15-PGDH structures. **a,** View of the cofactor binding pocket after superposition of the cryo-EM structure of the 15-PGDH-NADH-SW209415 complex with the 15-PGDH-NAD+ crystal structure (PDB entry: 2GDZ). Residues contacting NAD+ in the crystal structure are shown in dark green, while residues contacting NADH in the (+)-SW209415-bound structure are shown in orange. Residues contacting both NADH and (+)-SW209415 are shown in pink, and residues contacting (+)-SW209415 are shown in cyan. **b,** Electrostatic potential surfaces of NAD+ (left) and NADH (right). Blue and red represent positive and negative potentials (units of kT/e from -20 to +20).

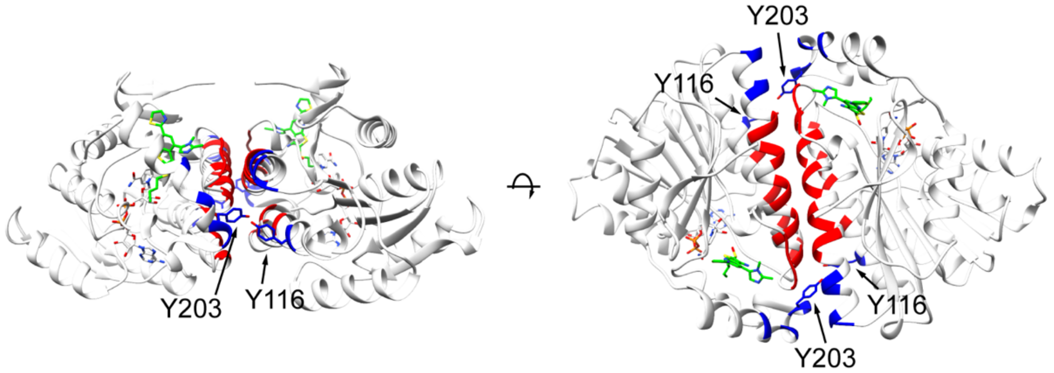

Supplementary Fig. 8 | Classification of the dimer interface based on the structural information of the 15-PGDH-NADH complex with and without (+)-SW209415 bound. Core dimer interfaces (colored in red) form even in the absence of inhibitor (+)-SW209415 (colored in green), while inducible dimer interfaces (colored in blue) form only upon binding of (+)-SW209415.

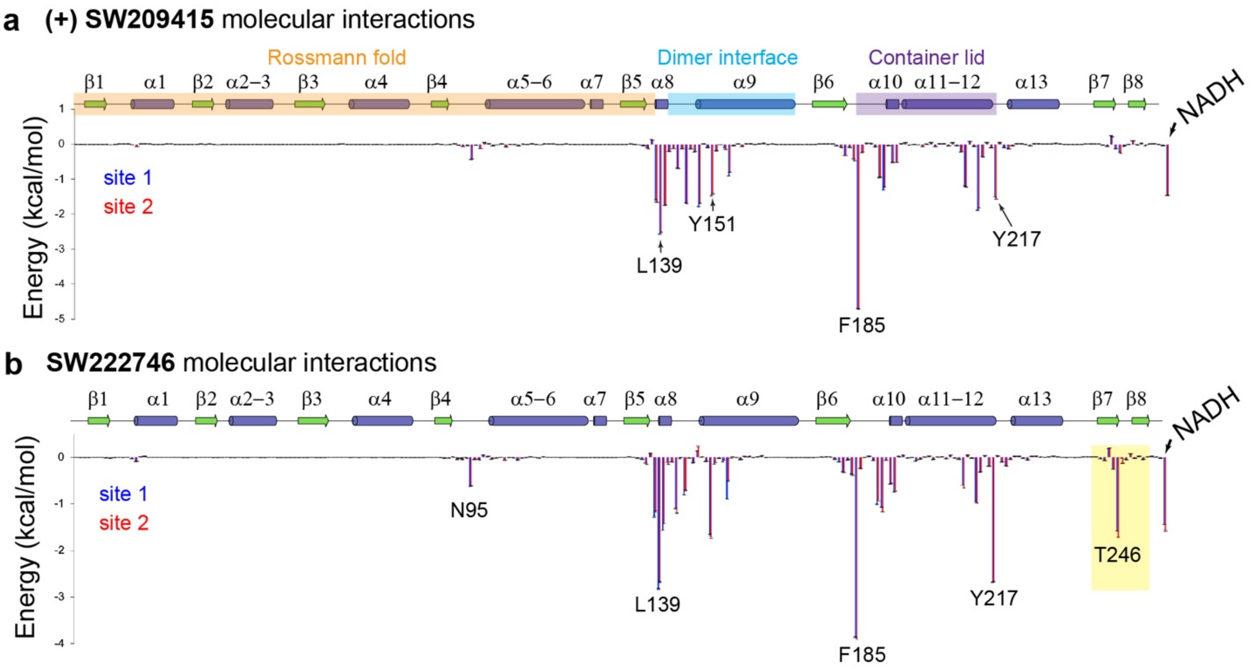

Supplementary Fig. 9 | Decomposition of binding energy on a per-residue basis for binding of two diverse inhibitors with 15-PGDH. Interactions are shown, as labeled, for 15-PGDH residue interactions with (+)-SW209415 (top) and SW222746 (bottom). In each case, drug binding sites for both protomers (site 1, blue bars; site 2, red bars) within the homodimer are shown to exhibit similar “residue fingerprint profiles”. More contributions from β7-8, including T246, of 15-PGDH are observed for SW222746 binding.

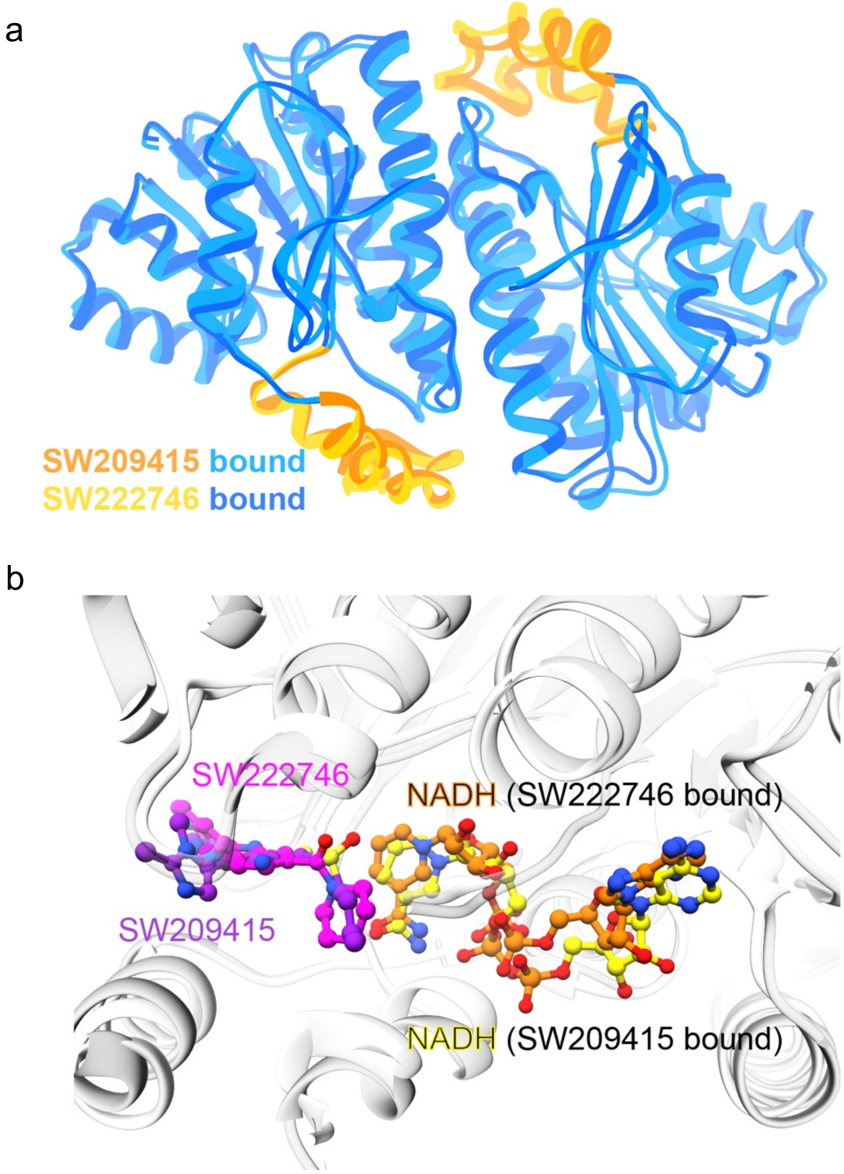

Supplementary Fig. 10 | Structural comparison of 15-PGDH-NADH homodimeric complex when bound to (+)-SW209415 or SW222746. **a,** Superposition of (+)-SW209415 and SW222746-bound human 15-PDGH structures. The structures were aligned to the protomer on the left side, showing that the protomer on the right side is differentially displaced due to binding of different chemical inhibitors. **b,** Superpositioning of the (+)-SW209415 and SW222746-bound 15-PGDH complexes reveals a shift in NADH cofactor positioning with respect to the bound inhibitor.

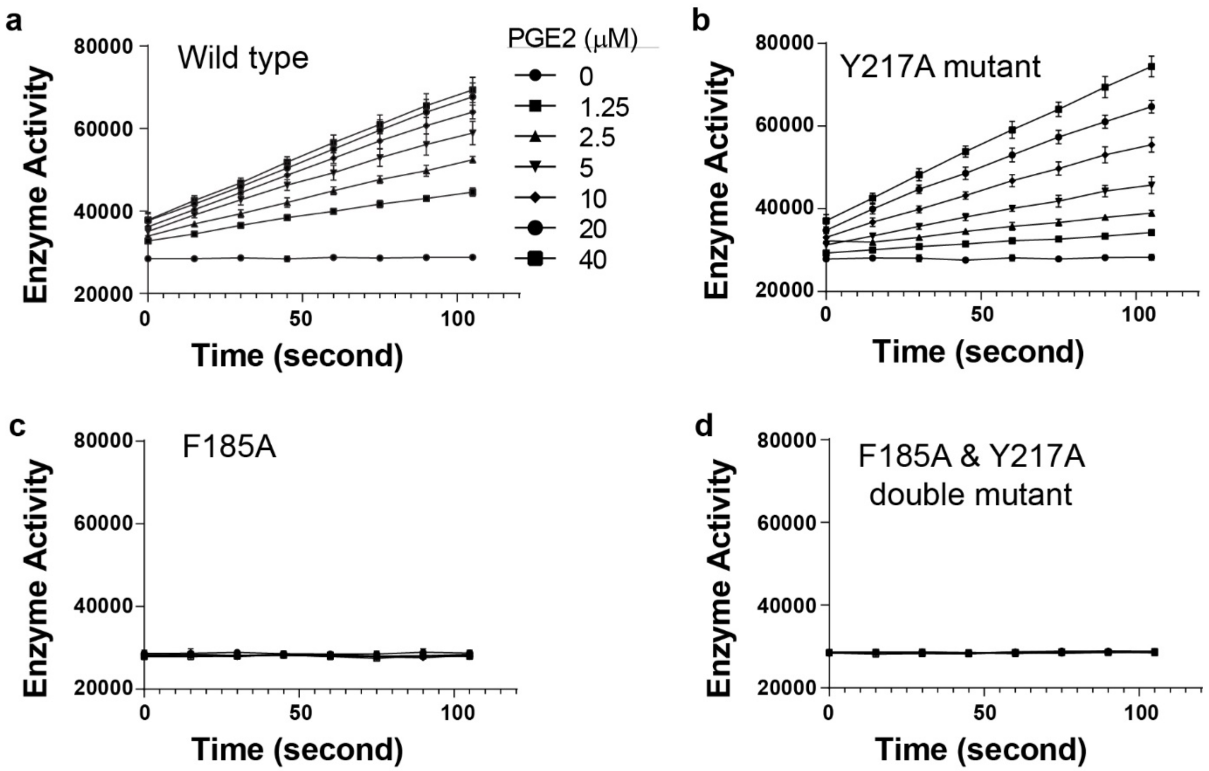

Supplementary Fig. 11 | Representative raw graphs for enzyme activity assay for 15-PGDH proteins. **a**, wild type **b**, Y217A mutant, **c**, F185A mutant and **d**, F185A + Y217A double mutant. Data are presented as mean values +/- SD (n=3).

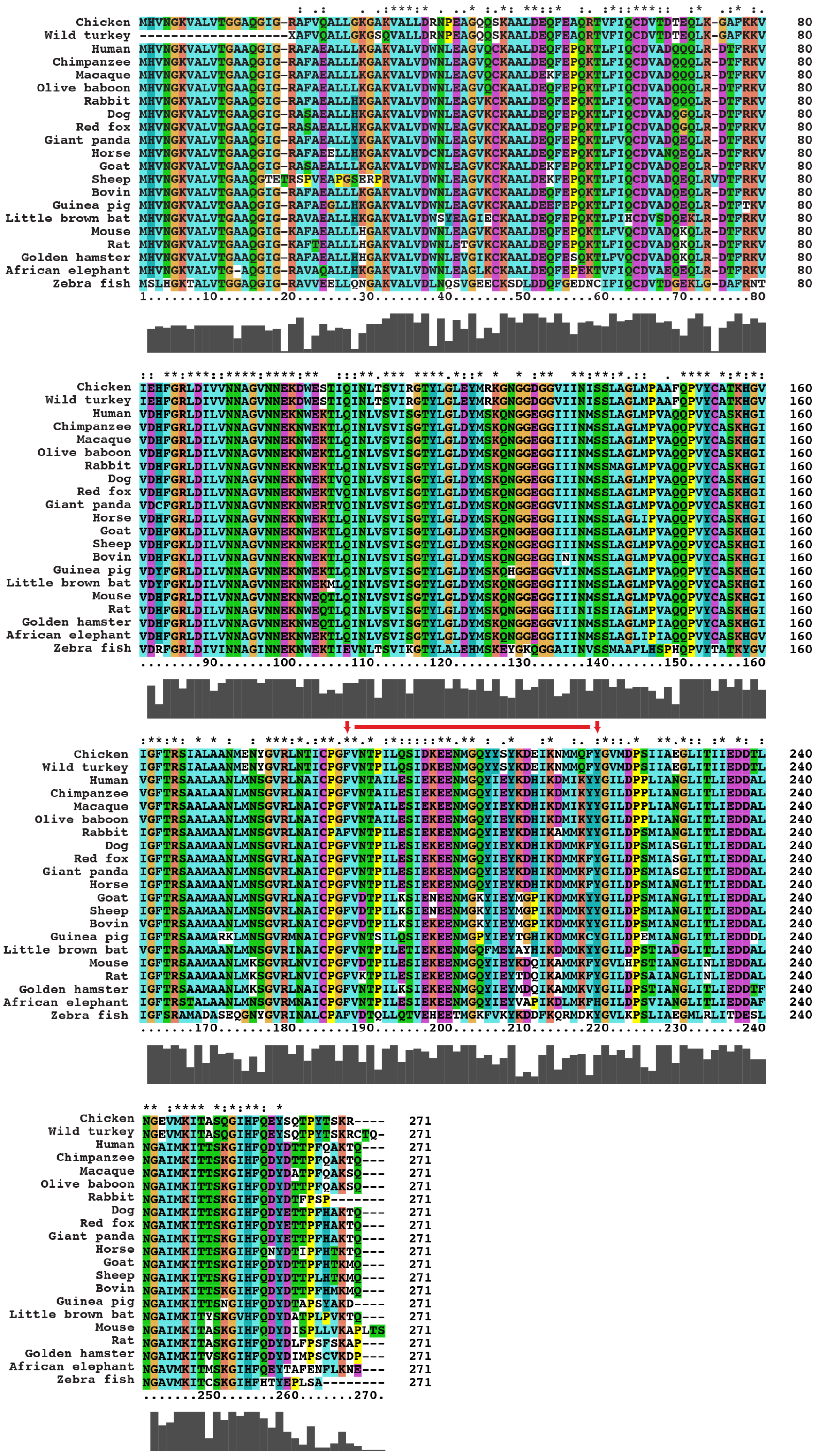

Supplementary Fig. 12 | Multiple sequence alignment of 15-PGDH protein from different species. F185 and Y217 are highlighted by red arrows, the lid consists of sequences in between these two residues.

Supplementary Table 1. The modification rates (k_i^{fp}) of hydroxyl radical footprinting and protection factors ($lnPF$) of individual residues in human 15-PGDH with and without (+)-SW209415 visualized by mass spectrometry. Residues with significant difference between the apo and bound state are highlighted in yellow.

Residue	k_{HRF}^{apo}	k_{HRF}^{415}	$lnPF^{apo}$	$lnPF^{415}$	$\Delta lnPF$
V10	0.02 ± 0.0009	0.017 ± 0.0012	1.188	1.486	0.298
Q15	0.019 ± 0.0013	0.026 ± 0.0026	0.192	0.014	-0.178
I17	0.044 ± 0.0021	0.018 ± 0.002	1.250	2.279	1.029
R19	0.027 ± 0.006	0.025 ± 0.0018	1.331	1.544	0.213
F21	0.017 ± 0.0012	0.018 ± 0.0016	3.136	3.214	0.078
E23	0.024 ± 0.0058	0.033 ± 0.004	0.003	-0.180	-0.183
L27	0.34 ± 0.038	0.33 ± 0.033	-0.793	-0.627	0.166
K28	0.047 ± 0.0057	0.055 ± 0.0062	0.500	0.478	-0.022
W37	2.16 ± 0.3	2.88 ± 0.026	-1.268	-1.420	-0.152
L39	0.35 ± 0.035	0.44 ± 0.0065	-0.822	-0.915	-0.093
E40	0.038 ± 0.0042	0.049 ± 0.0056	-0.456	-0.575	-0.119
E40	0.064 ± 0.014	0.09 ± 0.0015	-0.978	-1.183	-0.205
V43	0.082 ± 0.011	0.094 ± 0.0016	-0.223	-0.224	-0.001
C45	0.038 ± 0.0042	0.049 ± 0.0056	3.290	3.172	-0.118
K46	0.064 ± 0.014	0.09 ± 0.0015	0.191	-0.014	-0.205
F60	0.12 ± 0.012	0.15 ± 0.0051	1.182	1.094	-0.088
I61	0.14 ± 0.0086	0.17 ± 0.0021	0.092	0.034	-0.058
Q62	0.031 ± 0.0014	0.05 ± 0.0035	-0.297	-0.640	-0.343
C63	0.019 ± 0.0014	0.02 ± 0.0014	3.983	4.068	0.085
Y116	3.24 ± 0.28	0.56 ± 0.044	-2.046	-0.155	1.891
L117	2.52 ± 0.29	0.6 ± 0.054	-2.796	-1.225	1.571
I181	0.0079 ± 0.0007	0.0085 ± 0.0006	2.967	3.029	0.062
* C182	0.14 ± 0.021	0.18 ± 0.036	1.986	1.871	-0.115
* C182	0.032 ± 0.0014	0.016 ± 0.0026	3.462	4.291	0.829
P183	0.035 ± 0.0046	0.014 ± 0.0011	-0.003	1.049	1.052
F185	0.054 ± 0.0061	0.018 ± 0.0022	1.980	3.214	1.234
V186	0.07 ± 0.0058	0.028 ± 0.0035	-0.065	0.987	1.052
N187	0.01 ± 0.00064	0.0093 ± 0.0006	0.429	0.637	0.208
I190/L191	0.028 ± 0.002	0.0097 ± 0.0003	1.702	2.897	1.195
M213	107.32 ± 16.35	112.69 ± 2.08	-5.009	-4.923	0.086
Y217	0.23 ± 0.036	0.14 ± 0.014	0.599	1.231	0.632
D221	0.041 ± 0.0026	0.054 ± 0.0064	-1.029	-1.169	-0.14
P223	0.13 ± 0.008	0.16 ± 0.0066	-1.315	-1.387	-0.072

L224	0.3 ± 0.0022	0.45 ± 0.0045	-0.668	-0.937	-0.269
L229	0.018 ± 0.0057	0.02 ± 0.001	2.146	2.176	0.03
I230	0.019 ± 0.0095	0.021 ± 0.003	2.089	2.125	0.036
T231	0.069 ± 0.0077	0.067 ± 0.0052	-0.187	-0.022	0.165
Y256	27.3 ± 6.18	27.56 ± 6.45	-4.178	-4.051	0.127

* C182 has two different protection values that were calculated for residues labeled with one (+16) and two (+32) oxygens. C182 was predominantly labeled with one oxygen (+16), which is listed first in the Table.

Supplementary Table 2. Cryo-EM data collection, processing and model refinement statistics for human 15-PGDH and inhibitor-bound structures.

	15-PGDH with (+)-SW209415	apo 15-PGDH	15-PGDH with SW222746
Microscope	Titan Krios	Titan Krios	Titan Krios
Voltage (keV)	300	300	300
Image filter	BioQuantum	BioQuantum	BioQuantum
Slit width (eV)	20	20	20
Super-resolution Pixel size (Å)	0.26	0.26	0.25
Symmetry	C1	C1	C1
Defocus range (μm)	-0.5 to -2.5	-0.5 to -2.5	-0.5 to -2.5
Electron dose ($e^-/Å^2$)	108	108	110
Micrographs	8,371	10,248	5,784
Number of particles	776,110	267,049	722,732
Map resolution at 0.143 FSC (Å)	2.4	3.3	2.9
B-factor	68.6	119	70.4
Model Refinement			
Atom	8,110	3,425	8,050
Residues	Protein: 512	Protein: 444	Protein: 512
CCmask	0.85<br>0.88 (ligand)	0.61<br>0.65 (ligand)	0.80<br>0.82 (ligand)
Resolution (FSC map vs. model at 0.5) (Å)	2.5	4.1	3.4
r.m.s deviations			
Bond lengths (Å)	0.003	0.002	0.003
Bond angles (°)	0.627	0.491	0.765
Clash score	2.23	10.04	8.84
MolProbity score	1.00	2.15	1.59
Ramachandran plot (%)			
Allowed	1.77	2.98	2.57
Favored	98.23	97.02	97.43
PDB/EMDB IDs	8CVN/27010	8FD8/29005	8CWL/27025

Supplementary Table 3. Summary of MD simulations performed using Anton2

Starting Coordinates	Ligand	Cofactor	Total trajectory time (μs)
(+)-SW209415 bound	415	NADH	1.5 μs
apo	-	NADH	4.5 μs
SW222746 bound	746	NADH	1.5 μs
