# Supplementary material for: Small molecule inhibitors of 15-PGDH exploit a physiologic induced-fit closing system
Source: Nat Commun. 2023 Feb 11;14:784. doi: 10.1038/s41467-023-36463-7 (PMC9922282; doi:10.1038/s41467-023-36463-7)
Supplement: Supplementary file 3 — Description of Additional Supplementary Files [file 41467_2023_36463_MOESM3_ESM.pdf]

Supplementary Movie. 1 | The movie summarizes and describes the proposed mechanism and relationship of 15-PGDH inhibitor binding and closing of the protein lid. In the absence of inhibitor, the 15-PGDH lid is disordered. When inhibitor binds, hydrophobic interactions between it and the lid hinge residues, F185 and Y217, promote closing and ordering of the triple-helix lid and help to maintain it in an ordered and closed form.
