# Supplementary material for: Small molecule inhibitors of 15-PGDH exploit a physiologic induced-fit closing system
Source: Nat Commun. 2023 Feb 11;14:784. doi: 10.1038/s41467-023-36463-7 (PMC9922282; doi:10.1038/s41467-023-36463-7)
Supplement: Supplementary file 5 — Reporting Summary [file 41467_2023_36463_MOESM5_ESM.pdf]

nature portfolio

Corresponding author(s): Derek J. Taylor, Sanford Markowitz, Joseph Ready

Last updated by author(s): 06/20/2022

Reporting Summary

Nature Portfolio wishes to improve the reproducibility of the work that we publish. This form provides structure for consistency and transparency in reporting. For further information on Nature Portfolio policies, see our Editorial Policies and the Editorial Policy Checklist.

Statistics

For all statistical analyses, confirm that the following items are present in the figure legend, table legend, main text, or Methods section.

n/a	Confirmed	
☐	☒	The exact sample size (*n*) for each experimental group/condition, given as a discrete number and unit of measurement
☐	☒	A statement on whether measurements were taken from distinct samples or whether the same sample was measured repeatedly
☒	☐	The statistical test(s) used AND whether they are one- or two-sided<br>*Only common tests should be described solely by name; describe more complex techniques in the Methods section.*
☒	☐	A description of all covariates tested
☐	☒	A description of any assumptions or corrections, such as tests of normality and adjustment for multiple comparisons
☒	☐	A full description of the statistical parameters including central tendency (e.g. means) or other basic estimates (e.g. regression coefficient) AND variation (e.g. standard deviation) or associated estimates of uncertainty (e.g. confidence intervals)
☒	☐	For null hypothesis testing, the test statistic (e.g. *F*, *t*, *r*) with confidence intervals, effect sizes, degrees of freedom and *P* value noted<br>*Give P values as exact values whenever suitable.*
☒	☐	For Bayesian analysis, information on the choice of priors and Markov chain Monte Carlo settings
☒	☐	For hierarchical and complex designs, identification of the appropriate level for tests and full reporting of outcomes
☒	☐	Estimates of effect sizes (e.g. Cohen's *d*, Pearson's *r*), indicating how they were calculated

Our web collection on statistics for biologists contains articles on many of the points above.

Software and code

Policy information about availability of computer code

Data collection	CryoEM data was collected using SerialEM 3.7 (at CWRU) and Leginon (at Midwest Consortium for High-resolution Cryo-Electron Microscopy). Microsecond scale simulations were performed using Anton 2 at Pittsburgh Supercomputing Center. Protein thermal shift assays were performed using Perkin Elmer Envision Reader. Hydroxyl radical footprinting experiments were performed at beamline X28C of NSLS at BNL. More detailed information can be found in the Methods section.
Data analysis	Figures and movies were rendered using Chimera and ChimeraX 1.3; cryoEM data was analyzed using CryoSparc (v2.14.3-beta & v3.2.0) and Relion 3.1; atomic models were refined using Phenix 1.20.1; atomic models were manually adjusted in Coot 0.9.6; structure models are validated using MolProbility 4.5.1 and Q-scores were calculated using MapQ plugin in Chimera. Protein thermal shift assays were analyzed using GraphPad Prism 9.4.0. MD simulation trajectories were analyzed using VMD 1.9.4 and AMBER18. Hydroxyl radical footprinting experiments were analyzed using Mass Matrix and Origin 8.0 for rate quantification. More detailed information can be found in the Methods section.

For manuscripts utilizing custom algorithms or software that are central to the research but not yet described in published literature, software must be made available to editors and reviewers. We strongly encourage code deposition in a community repository (e.g. GitHub). See the Nature Portfolio guidelines for submitting code & software for further information.

Data

Policy information about availability of data

All manuscripts must include a data availability statement. This statement should provide the following information, where applicable:

- Accession codes, unique identifiers, or web links for publicly available datasets
- A description of any restrictions on data availability
- For clinical datasets or third party data, please ensure that the statement adheres to our policy

The cryo-EM maps of (+)-SW209415 and SW222746 bound to 15-PGDH with NADH and the apo-form with NADH have been deposited into the Electron Microscopy Data Bank under accession codes EMD-27010, EMD-27025 and EMD-29005, respectively. The corresponding atomic models have been deposited into Protein Data Bank under accession codes 8CVN, 8CWL and 8FD8. All maps and models will be released with publication.

Human research participants

Policy information about studies involving human research participants and Sex and Gender in Research.

Reporting on sex and gender	n/a
Population characteristics	n/a
Recruitment	n/a
Ethics oversight	n/a

Note that full information on the approval of the study protocol must also be provided in the manuscript.

Field-specific reporting

Please select the one below that is the best fit for your research. If you are not sure, read the appropriate sections before making your selection.

☒ Life sciences ☐ Behavioural & social sciences ☐ Ecological, evolutionary & environmental sciences

For a reference copy of the document with all sections, see nature.com/documents/nr-reporting-summary-flat.pdf

Life sciences study design

All studies must disclose on these points even when the disclosure is negative.

Sample size	~~No sample size calculations were performed.~~
Data exclusions	In hydroxyl radical footprinting protection factor analysis, modified peptides without single amino acid resolution were not used for the analysis.
Replication	Protein thermal shift assays and enzyme kinetics were repeated at least three times for each sample. Hydroxyl radical protein footprinting experiments were repeated twice.
Randomization	No samples were allocated to different experimental groups for assessment of alternative approaches, treatments or experimental analysis. So this aspect of study design is not relevant.
Blinding	Blinding is not directly relevant, however, individual experiments were performed in three different labs independently.

Reporting for specific materials, systems and methods

We require information from authors about some types of materials, experimental systems and methods used in many studies. Here, indicate whether each material, system or method listed is relevant to your study. If you are not sure if a list item applies to your research, read the appropriate section before selecting a response.

Materials & experimental systems

n/a	Involved in the study
☒	☐ Antibodies
☒	☐ Eukaryotic cell lines
☒	☐ Palaeontology and archaeology
☒	☐ Animals and other organisms
☒	☐ Clinical data
☒	☐ Dual use research of concern

Methods

n/a	Involved in the study
☒	☐ ChIP-seq
☒	☐ Flow cytometry
☒	☐ MRI-based neuroimaging
